# Associations between birth registration and early child growth and development: evidence from 31 low- and middle-income countries

**DOI:** 10.1186/s12889-018-5598-z

**Published:** 2018-05-30

**Authors:** Joshua Jeong, Amiya Bhatia, Günther Fink

**Affiliations:** 1 Department of Global Health and Population, Harvard T.H. Chan School of Public Health, 665 Huntington Avenue, 11th floor, Boston, MA USA; 2 Department of Social and Behavioral Sciences, Harvard T.H. Chan School of Public Health, Boston, USA; 3 Swiss Tropical and Public Health Institute, Basel, Switzerland; 4 University of Basel, Basel, Switzerland

**Keywords:** Birth registration, Early child nutrition, Early child development, Multiple Indicator cluster surveys, Low- and middle-income countries

## Abstract

**Background:**

Lack of legal identification documents can impose major challenges for children in low- and middle-income countries (LMICs). The aim of this study was to investigate the association between not having a birth certificate and young children’s physical growth and developmental outcomes in LMICs.

**Methods:**

We combined nationally representative data from the Multiple Indicator Cluster Surveys in 31 LMICs. For our measure of birth registration, primary caregivers reported on whether the child had a birth certificate. Early child outcome measures focused on height-for-age z-scores (HAZ), weight-for-age z-scores (WAZ), weight-for-height z-scores (WHZ), and standardized scores of the Early Childhood Development Index (ECDI) for a subsample of children aged 36–59 months. We used linear regression models with country fixed effects to estimate the relationship between birth registration and child outcomes. In fully adjusted models, we controlled for a variety of child, caregiver, household, and access to child services covariates, including cluster-level fixed effects.

**Results:**

In the total sample, 34.7% of children aged 0–59 months did not possess a birth certificate. After controlling for covariates, not owning a birth certificate was associated with lower HAZ (β = − 0.18; 95% CI: -0.23, − 0.14), WAZ (β = − 0.10, 95% CI: -0.13, − 0.07), and ECDI z-scores (β = − 0.10; 95% CI: -0.13, − 0.07) among children aged 36–59 months.

**Conclusion:**

Our findings document links between birth registration and children’s early growth and development outcomes. Efforts to increase birth registration may be promising for promoting early childhood development in LMICs.

**Electronic supplementary material:**

The online version of this article (10.1186/s12889-018-5598-z) contains supplementary material, which is available to authorized users.

## Background

The United Nations Convention on the Rights of the Child entitles every child to be registered immediately after birth [1]. Birth registration, an important measure of legal identity, is recognized in target 16.9 of the Sustainable Development Goals (SDGs), which aims to “provide legal identity for all, including birth registration” by 2030 [2]. Yet, globally nearly 230 million children under-5 have never been officially been registered [3], or formally recognized by the state [4].

Ensuring that all children survive and thrive, receive good health care and education, and have equal chances to achieve their full developmental potential during their early years are also key pillars of the SDGs [5, 6]. More specifically, the SDGs prioritize reducing malnutrition (target 2.2) for the estimated 155 million children under-5 globally who were stunted in 2016 [7], and promoting early childhood development (ECD; target 4.2) for the estimated 250 million children under-5 globally at risk of poor development [2, 8]. Over the past decade, several studies have attempted to determine the key risk factors and correlates of child undernutrition and poor development outcomes in low- and middle-income countries (LMICs), generally highlighting the importance of fetal growth, poverty, poor water and sanitation, as well as inadequate home environments [9–11]. However, the role of birth registration has been largely absent in these prior global reviews on correlates of early child nutrition and development outcomes.

Lack of birth registration violates children’s fundamental rights, including their right to nationality, and may also hinder young children’s access to targeted health services and social welfare programs (e.g. cash transfer schemes) and enrollment in school [12–17]. Without a birth certificate, a child's exact age is challenging to prove, which is important for ensuring that children receive age-appropriate recommended schedule of vaccinations [14], applying correct growth standards to estimate children's nutritional status (i.e., height-for-age or weight-for-age) [18, 19], and verifying that children are at least a minimum age upon entering school. As children grow older, a birth certificate can provide important documentation in protecting against child labor, trafficking, and sexual exploitation [1, 12]; all which are associated with poor child health and wellbeing outcomes [20, 21]. Moreover, unregistered children are not counted and thus excluded from civil registration systems, which provide governments with vital statistics for allocating resources and monitoring programs and policies that have direct implications for children’s nutrition and development [22].

To date, much of the global literature on birth registration has been at a macro-level: arguing principally from a rights-based legal approach and emphasizing the normative importance of birth registration [23], or advocating for the importance of civil registration and vital statistics systems [24, 25]. A growing body of evidence has identified predictors of birth registration in order to develop strategies for increasing birth registration coverage [13, 26–28]. While a few studies to date have examined associations between birth registration and children’s early nutrition and growth outcomes in LMICs [12, 18], no study known to the authors has additionally explored the association between birth registration and ECD outcomes in LMICs. Given existing research on the importance of birth registration, we hypothesized that not being registered would be negatively associated with early childhood growth and development outcomes in LMICs.

## Methods

### Data

We used data from UNICEF’s Multiple Indicator Cluster Survey (MICS), an international household survey program that collects information about the health, nutrition, education, and development of children in LMICs. The MICS is unique for collecting and monitoring ECD in a standardized and comparable way across LMICs, and remains the primary data source to measure and monitor ECD outcomes. We combined all nationally representative surveys from MICS rounds 4 and 5 (2010–2014) that were publicly available prior to January, 2017. We restricted our sample to children who had data on birth registration and either data on anthropometric outcomes or data on the Early Childhood Development Index (ECDI), which is primarily collected for preschool children aged 36–59 months.

### Outcomes

We examined four child outcomes relating to early nutrition and development: height-for-age z-scores (HAZ), weight-for-age z-scores (WAZ), weight-for-height z-scores (WHZ), and ECDI z-scores. Z-scores for anthropometric measures were computed using the 2006 WHO Multi-center Growth Reference Study standards [29]. Biologically implausible values (HAZ as <− 6 or > 6, WAZ as <− 6 or > 5, and WHZ as <− 5 or > 5) were excluded based on WHO cutoffs [30].

Early child development was measured using the ECDI. Developed by UNICEF for 3- and 4-year-olds surveyed in the MICS household survey program, the ECDI is comprised of 10 caregiver-reported, dichotomously-scored questions to assess 4 developmental domains: cognitive, socioemotional, literacy-numeracy, and physical development. These 10 items were determined through multi-country field tests, validity, and reliability studies, and deliberation with experts [31]. This population measure of ECD has been used in other recent studies [32, 33]. A composite score for ECD was created (ranging from 0 to 10) by summing the number of positive responses across the literacy-numeracy, social-emotional, learning, and physical domain items, and normalized to a ECDI z-score for direct comparability and ease of interpretation to the standardized scale of HAZ, WAZ, and WHZ.

### Independent variable

Our primary independent variable of interest was lack of a birth certificate. In the MICS questionnaire, two items directly capture birth registration: first, caregivers are asked to show the interviewer the child’s birth certificate. If a birth certificate is not available, caregivers are asked whether the child ever had a birth certificate, and if not, whether the child’s birth had been registered with the civil authorities. For our empirical analysis, we created a no birth certificate indicator variable, which was coded 1 if the child did not ever have a birth certificate and 0 if the child currently had or previously had a birth certificate.

### Covariates

We adjusted for a variety of child-, caregiver-, and household-level covariates. Child characteristics included age (in months) and sex (male or female). Caregiver characteristics included maternal and paternal highest level of education (no formal education, primary, or secondary or higher), maternal age (5-year age categories from 15 to 49 years), and maternal marital status (currently married, formerly married, or never married). Household characteristics included household wealth index (quintiles within each country: calculated as a principal component of a group of assets owned by the household [34]) and place of residency (urban or rural). Utilization of child health and learning services was measured by the number of vaccinations received (ranging from 0 to 4 for bcg and at least one dose of dpt/hepb, polio, and measles) and whether or not preschool-aged children attended an early education program (asked only regarding children aged 36–59 months).

### Analysis

We conducted a complete case analysis upon verifying that missingness was not systematic. We specified a series of four linear regression models with varying controls for potential confounders and mediators to estimate the association between lack of birth registration and each of the four outcome variables of interest: HAZ, WAZ, WHZ, and ECDI z-score among children aged 36–59 months. Model 1 only adjusted for child age, sex, and country fixed effects. Model 2 further adjusted for all caregiver- and household-level demographic and socioeconomic covariates (maternal and paternal education, maternal age, maternal marital status, household wealth index, and place of residency). Model 3 further adjusted for variables representing utilization of services that may relate to both birth registration and ECD outcomes (children’s vaccinations and early childhood education programs). Finally, Model 4 additionally included primary sampling unit (PSU)/cluster-level fixed effects, which can account for other observable and unobservable differences in socioeconomic, environmental, and institutional characteristics of local enumeration areas that are common to all respondents from that area (i.e., local diet, community child health awareness campaigns, cultural and social norms, as well as within-cluster availability of birth registration and other social services). Standard errors across all models were clustered at the PSU-level to account for the complex MICS survey design. All analyses were conducted using Stata version 13 [35].

### Sensitivity analyses and robustness checks

First, to assess whether pooled findings were robust, we conducted separate country-specific models (fully adjusted Model 4) for the associations between lack of birth registration and each of the four child outcome variables; and employed random-effects meta-regressions to re-estimate a pooled effect that accounts for the varying sample sizes across country surveys. Second, to examine whether not having a birth certificate was related to child outcomes as early as in the first 3 years of life and whether the magnitude of these associations increased by child age (categorized in 12-month age groups) we re-specified Model 4, excluding early childhood education attendance as a utilization of service covariate. This allowed us to explore the associations between not having a birth certificate and children’s HAZ and WAZ outcomes in a separate sample of younger children aged 0–35 months, for whom anthropometric data, but not the ECDI, were available.

## Results

A total of 157,336 children aged 0 to 59 months from 31 countries were represented in the full sample. No significant differences were detected between the complete cases in the analytic sample and the incomplete cases (*N* = 65,425, 29.4% of original sample) that were excluded due to missing data on full covariates. Sample characteristics for the total sample of children are presented in Table 1. The average age of the child was 28 months, and nearly half of the sample was female. Overall, 28.5% of mothers and 18.4% of fathers reported no formal education. The majority of households (60.2%) resided in rural areas.

**Table 1:** Full sample characteristics among children aged 0–59 months and by children’s birth certificate ownership

	Total (*n* = 157,336)	Child does not have birth certificate (*n* = 54,605)	Child has birth certificate (*n* = 102,731)
	% (95% CI)	% (95% CI)	% (95% CI)
Covariates			
Female child	49.1 (48.9–49.4)	49.6 (49.2–50.0)	48.8 (48.5–49.1)
Age of child, mean (SD), range 0–59 months	28.0 (16.7)	25.3 (16.4)	29.4 (16.6)
Maternal education			
None	28.5 (27.9–29.1)	41.4 (40.4–42.4)	21.6 (21.1–22.2)
Primary	33.1 (32.7–33.6)	36.7 (35.9–37.4)	31.2 (30.7–31.8)
Secondary or higher	38.4 (37.8–39.0)	21.9 (21.2–22.6)	47.1 (46.5–47.8)
Paternal education			
None	18.4 (17.9–18.9)	28.3 (27.3–29.2)	13.2 (12.7–13.6)
Primary	30.7 (30.3–31.2)	35.4 (34.6–36.1)	28.3 (27.8–28.8)
Secondary or higher	50.8 (50.3–51.4)	36.4 (35.5–37.2)	58.5 (58.0–59.1)
Martial status			
Currently married/in union	99.8 (99.7–99.8)	99.7 (99.7–99.8)	99.8 (99.8–99.8)
Formerly married/in union	0.1 (0.1–0.1)	0.2 (0.1–0.2)	0.1 (0.1–0.1)
Never married/in union	0.1 (0.1–0.1)	0.1 (0.1–0.2)	0.1 (0.1–0.1)
Maternal age			
15–19	4.1 (4.0–4.2)	6.0 (5.7–6.2)	3.1 (3.0–3.2)
20–24	19.6 (19.3–19.9)	23.0 (22.5–23.4)	17.8 (17.5–18.2)
25–29	28.2 (27.9–28.5)	27.4 (27.0–27.9)	28.6 (28.2–28.9)
30–34	23.2 (22.9–23.5)	20.5 (20.1–20.9)	24.7 (24.3–25.0)
35–39	15.8 (15.6–16.1)	14.2 (13.8–14.6)	16.7 (16.4–17.0)
40–44	7.1 (7.0–7.3)	6.6 (6.4–6.9)	7.4 (7.2–7.6)
45–49	2.0 (1.9–2.0)	2.3 (2.2–2.5)	1.8 (1.7–1.9)
Wealth quintile			
Poorest	18.7 (18.2–19.2)	22.4 (21.7–23.1)	16.8 (16.2–17.3)
Poor	20 (19.6–20.3)	23.4 (22.8–24.0)	18.1 (17.7–18.5)
Middle	19.6 (19.3–19.9)	21.4 (20.9–22.0)	18.6 (18.2–19.0)
Rich	19.8 (19.5–20.2)	17.9 (17.3–18.5)	20.8 (20.4–21.3)
Richest	21.9 (21.4–22.4)	14.8 (14.2–15.5)	25.7 (25.1–26.2)
Rural residence	60.2 (59.4–61.0)	78.4 (77.3–79.5)	50.6 (49.6–51.5)
Number of vaccines child received, mean (SD), range 0–4	3.4 (0.8)	3.3 (0.9)	3.4 (0.8)
Child currently attends ECE, among children aged 36–59 months	24.5 (23.9–25.0)	16.3 (15.4–17.1)	27.5 (26.9–28.2)
Child outcomes			
HAZ, mean (SD)	−1.01 (1.7)	−1.49 (1.7)	−0.75 (1.7)
WAZ, mean (SD)	−0.58 (1.4)	−1.05 (1.4)	− 0.32 (1.3)
WHZ, mean (SD)	−0.01 (1.4)	− 0.29 (1.4)	0.15 (1.5)
ECDI z-scores among children aged 36–59 months, mean (SD)	0.00 (1.0)	−0.34 (0.9)	0.18 (1.0)

*CI* confidence interval, *ECE* early childhood education, *ECDI* Early Childhood Development Index, *HAZ* height-for-age z-scores, *SD* standard deviation, *WAZ* weight-for-age z-scores, *WHZ* weight-for-height z-scores

Approximately one in three children under-5 (34.7%) did not possess a birth certificate. The average proportion of children without a birth certificate varied across countries, ranging from as low 0.2 and 0.5% in Ukraine and Thailand, respectively, (where nearly all children were registered) to as high as 80.8 and 95.0% in Guinea Bissau and Malawi, respectively (Additional file 1). Children who did not have a birth certificate were more likely to have parents who were less educated, live in poorer households, and reside in rural areas of the country (Table 1).

The mean HAZ was − 1.01 (SD = 1.7), with 26.2% of children classified as stunted. The mean WAZ for children was − 0.58 (SD = 1.4), with 13.7% of infants exhibiting underweight. The mean WHZ for children was − 0.01 (SD = 1.4), with 7.0% classified as wasted. The mean ECDI z-score was 0.00 (SD = 1.0).

Of this total sample, we primarily focused on 54,916 children aged 36 to 59 months in 24 LMICs, for whom full information was available across all variables of interest. Table 2 presents adjusted associations between a lack of birth certificate and preschool-aged children’s nutrition and development outcomes across the four model specifications. In models only adjusting for child age, sex, and country-level fixed effects (Model 1), not having a birth certificate was negatively associated with children’s HAZ (β = − 0.48; 95% CI: -0.52, − 0.43), WAZ (β = − 0.30; 95% CI: -0.33, − 0.27), and ECDI z-scores (β = − 0.32; 95% CI: -0.34, − 0.29); associations however were not significant for WHZ (β = − 0.01; 95% CI: -0.04, 0.03). In models additionally adjusting for caregiver and sociodemographic covariates (Model 2), associations were smaller in magnitude but remained significant for HAZ (β = − 0.26; 95% CI: -0.30, − 0.22), WAZ (β = − 0.15; 95% CI: -0.18, − 0.11), and ECDI z-scores (β = − 0.15; 95% CI: -0.18, − 0.13). In models additionally adjusting for children’s utilization of health and education services (Model 3), associations were further attenuated for HAZ (β = − 0.21; 95% CI: -0.25, − 0.17), WAZ (β = − 0.11; 95% CI: -0.14, − 0.08), and ECDI z-scores (β = − 0.10; 95% CI: -0.13, − 0.08). Finally, in models additionally accounting for PSU/cluster-level fixed effects (Model 4), significant negative associations persisted between not having a birth certificate and children’s HAZ (β = − 0.18; 95% CI: -0.23, − 0.14), WAZ (β = − 0.10, 95% CI: -0.13, − 0.07), and ECDI z-scores (β = − 0.10; 95% CI: -0.13, − 0.07).

**Table 2:** Associations between no birth certificate and children’s HAZ, WAZ, WHZ, and ECDI z-scores among children aged 36–59 months

Child outcomes	Model 1	Model 2	Model 3	Model 4
HAZ (*n* = 50,291)				
β of no birth certificate	−0.48***	−0.26***	− 0.21***	−0.18***
95% CI	(−0.52, − 0.43)	(− 0.30, − 0.22)	(−0.25, − 0.17)	(−0.23, − 0.14)
WAZ (*n* = 50,531)				
β of no birth certificate	− 0.30***	−0.15***	− 0.11***	−0.10***
95% CI	(−0.33, − 0.27)	(− 0.18, − 0.11)	(−0.14, − 0.08)	(−0.13, − 0.07)
WHZ (*n* = 50,178)				
β of no birth certificate	− 0.01	0.02	0.03	0.02
95% CI	(−0.04, 0.03)	(−0.01, 0.06)	(− 0.00, 0.07)	(− 0.01, 0.06)
ECDI z-score (*n* = 54,861)				
β of no birth certificate	− 0.32***	−0.15***	− 0.10***	−0.10***
95% CI	(−0.34, − 0.29)	(− 0.18, − 0.13)	(−0.13, − 0.08)	(−0.13, − 0.07)

*CI* confidence interval, *ECDI* Early Childhood Development Index, *HAZ* height-for-age z-scores, *WAZ* weight-for-age z-scores, *WHZ* weight-for-height z-scores

The table presents unweighted standardized mean differences in child growth and development outcomes for children aged 36–59 months in 31 countries who did not have a birth certificate. Model 1 only adjusted for child age, sex, and country-level fixed effects. Model 2 further adjusted for maternal age, maternal education, paternal education, household wealth quintiles, and urban/rural residency. Model 3 additionally adjusted for vaccinations and attendance in an early childhood education program. Model 4 additionally adjusted for primary sampling unit-level fixed effects. All standard errors were clustered at the primary sampling unit level. ***P-value < 0.001

### Sensitivity analyses and robustness checks

Overall pooled estimates based on meta-regression (using Model 4) were robust and comparable in magnitude to findings from pooled analyses (presented above in Table 2) for all outcomes. Significant relationships were found for children’s HAZ (Additional file 2), WAZ (Additional file 3), and ECDI (Additional file 4); associations for WHZ (Additional file 5) were not significant. While country specific results highlighted variation in the associations across countries, point estimates were largely consistent in magnitude and directionality across countries for each outcome. Of note, three countries (Lebanon, Macedonia and Moldova) were exceptions, which also had the smallest sample sizes and where only less than 4% of children did not have birth certificates.

In a separate sample of younger children aged 0–35 months (*N* = 102,488), the overall associations between a lack of birth certificate and children’s HAZ and WAZ were smaller in magnitude, but also remained significant (Additional file 6). In addition, findings indicated that the magnitude of these associations increased with child age: such that associations were strongest for 2-year-olds (as compared to 1-year-olds or children under-1).

## Discussion

Using data from 31 LMICs, our study revealed two main findings. First, birth registration among the children under-5 was low in our pooled sample. Despite the recommendation for birth registration to occur in the first few weeks or months of life, one in three children under-5 in the total sample were still without a birth certificate. We also found inequalities in access to birth certificates by wealth, maternal and paternal education and rural residency. This is consistent with other global studies of birth registration that have described large gaps in access to birth registration [36, 37].

Second, we found that not having a birth certificate was negatively associated with both preschool-aged children’s growth and developmental outcomes, or more specifically HAZ, WAZ, and ECDI z-scores. Our findings build upon the results of a prior study by Comandini et al. [18] that documented a negative relationship between birth registration and undernutrition among children aged 2-to-5 years in 37 sub-Saharan African countries. In our study, we newly highlight associations between birth registration and ECD, as indexed by the ECDI; and with respect to both child growth and development outcomes, even after adjusting for additional covariates than considered in prior research.

We did not find significant associations between birth registration and WHZ. One explanation could be the fact that WHZ calculations do not require information on age, which has been highlighted as more likely introducing bias and error among unregistered children and driving underestimations of their undernutrition status [18]. Another likely explanation for the null association with WHZ could be the fact that wasting is an indicator of acute malnutrition, often occurring suddenly due to contemporaneous shocks, such as infection or famine, and largely explained by dietary diversity, food insecurity, and climate change [38, 39]. Moreover, the prevalence of wasting in our sampled countries was very low (mean of 7.0%, with prevalence of wasting > 10% in only 3 countries) and potentially too small to detect differences.

With respect to the likely mechanisms, we found that socioeconomic factors (i.e., maternal and paternal education, household wealth index, and place of residency) explained nearly half of the unadjusted associations between birth registration and poor early child growth and development outcomes. Above and beyond household socioeconomic factors, children’s utilization of early health and learning services (i.e., child vaccinations and attendance in an early education programs) explained approximately a quarter to a third of the remaining association between birth registration and child outcomes. Interestingly, we found that additionally controlling for cluster characteristics did not add explanatory power, thereby minimizing the possibility that these associations are due to community characteristics within clusters within countries. Prior studies have also documented links between birth registration and children’s healthcare utilization, school enrollment and completion, and participation in social services (e.g., cash transfer programs and government food programs) [12–14]. Our findings extend this evidence by demonstrating how such services do, in turn, explain a considerable proportion of the direct associations between birth registration and early child nutrition and development outcomes.

However, we found that significant associations persisted between birth registration and child outcomes, which were unexplained by the covariates and cluster-fixed effects included in our models. One possible explanation could be that registration reflects some degree of parental investment in the child. If completing the registration process is arduous, parents who register their children may be those who have more time or financial resources that they are able to spend on their children (especially if registration involves traveling a distance or financial and opportunity costs) [40–42], or those who are more motivated and committed to following through with formal registration application procedures [43]. Another possibility could be that birth registration reflects a household’s social connectedness or social status (which could be shaped by sociodemographic factors that we do not include, e.g. ethnicity, caste, or religion) which may serve as a proxy for marginalization or how informed or empowered parents may be feel in accessing a range of formal and informal services. Future research that investigates parental knowledge and attitudes regarding birth registration and more comprehensively assesses the linkages between birth registration and a wider range of social services may better elucidate the factors that underlie our exploratory findings.

While our results support robust associations in this pooled sample, it is important to note that the opportunities afforded by a birth certificate vary considerably across country contexts. For instance, in Vietnam a birth certificate is necessary to enroll in both preschool and primary school, while in Sierra Leone and India, national policy mandates that birth certificates are not formally required at any stage of the education system [44]. Moreover, requiring a certificate to access services may disproportionately impact the most vulnerable groups within-countries [45]. Future research on birth registration should consider a country’s legal and policy environment and examine how these associations with early child outcomes are similar or different within and across LMICs and the proportion of variance explained at the country-level (e.g., using multilevel models).

Despite these policy differences across countries, our pooled findings indicate a significant negative relationship between birth registration and child growth and development outcomes across LMICs, and the potential role of socioeconomic factors in explaining a part of this relationship. This suggests important links between birth registration, social protection, and early child health and education services, especially for the children living in the poorest households. Research from Ghana has affirmed the benefits of incorporating birth registration into community health care and child health campaigns [40]. Moreover, the recent Lancet ECD series has emphasized the need for multisectoral approaches to coordinating ECD programs, particularly with the health and nutrition sectors [46, 47]. However, most ECD interventions and policies do not include birth registration as a core component [12–18]. Future efforts should consider integrating birth registration campaigns with other early childhood services and interventions to promote the development and well-being of young children.

Birth registration and the estimation of a child’s age is central to the very measurement of early childhood outcomes: a precise measurement of age is needed to accurately measure HAZ and WAZ among children under-5. Comandini and co-authors describe the negative effects of measurement error and age heaping in misestimating HAZ and WAZ, especially among children without a birth certificate [18, 19]. Efforts to improve birth registration could also address the processes of imputing, estimating, and guessing a child’s age in household surveys and improve the assessment of nutritional outcomes.

There are several limitations to this analysis. First, we were only able to pool data from countries for which the MICS data were available; therefore, our results may not be representative of LMICs as a whole. Second, although we adjusted for a range of covariates and include cluster-level fixed effects, we were unable to control for other important variables, such as data on facility birth, maternal autonomy, and access to other services. Third, both the ECDI and birth registration were caregiver reported and may be susceptible to recall bias. Fourth, measures of nutritional status (e.g. height or weight for age) could be prone to bias among unregistered children, as their age cannot be verified [18]. Finally, the MICS are cross-sectional surveys, which preclude causal interpretation, determination of mediators, and directionality of the associations.

## Conclusions

This study highlights gaps in birth registration for young children in LMICs and finds that not having a birth certificate is negatively associated with early child growth and development outcomes. Early child health, nutrition, and education programs and policies should consider integrating birth registration – and child protection more broadly – in order to ensure that every child is legally recognized and has a fair chance to achieve her full developmental potential.

## Additional files

Additional file 1: Country mean values for proportion of children not having a birth certificate, children’s HAZ, WAZ, WHZ, and ECDI z-score values among children aged 0–59 months. (DOCX 96 kb)

Additional file 2: Figure of pooled and country-specific associations between no birth certificate and HAZ among children aged 36–59 months based on meta-regression model. (DOCX 718 kb)

Additional file 3: Figure of pooled and country-specific associations between no birth certificate and WAZ among children aged 36–59 months based on meta-regression model. (DOCX 732 kb)

Additional file 4: Figure of pooled and country-specific associations between no birth certificate and ECDI z-score among children aged 36–59 months based on meta-regression model. (DOCX 749 kb)

Additional file 5: Figure of pooled and country-specific associations between no birth certificate and WHZ among children aged 36–59 months based on meta-regression model. (DOCX 715 kb)

Additional file 6: Sensitivity analysis of the associations between no birth certificate and HAZ and WAZ for children aged 0–35 months; and stratified by child age groups (12 month age groups). (DOCX 79 kb)

## Abbreviations

ECD: Early childhood development
ECDI: Early childhood development index
HAZ: Height-for-age z-scores
LMICs: Low- and middle-income countries
MICS: Multiple indicator cluster survey
PSU: primary sampling unit
SDGs: Sustainable development goals
WAZ: Weight-for-age z-scores
WHZ: Weight-for-height z-scores
