# Supplementary material for: Associations between birth registration and early child growth and development: evidence from 31 low- and middle-income countries
Source: BMC Public Health. 2018 May 30;18:673. doi: 10.1186/s12889-018-5598-z (PMC5977554; doi:10.1186/s12889-018-5598-z)
Supplement: Supplementary file 1 — Country mean values for proportion of children not having a birth certificate, children’s HAZ, WAZ, WHZ, and ECDI z-score values among children aged 0–59 months. (DOCX 96 kb) [file 12889_2018_5598_MOESM1_ESM.docx]

| **S1 Table. Country mean values for proportion of children not having a birth certificate, children’s HAZ, WAZ, WHZ, and ECDI z-score values among children aged 0-59 months** | | | | | |
| --- | --- | --- | --- | --- | --- |
| Country name | % of children not having a birth certificate | HAZ | WAZ | WHZ | ECDI  z-score |
| Ukraine | 0.2% |  |  |  | 0.58 |
| Thailand | 0.5% | -0.62 | -0.26 | 0.13 | 1.08 |
| Kazakhstan | 0.6% | -0.20 | 0.35 | 0.63 | 0.48 |
| Lebanon | 1.0% | -0.40 | 0.10 | 0.46 | 0.61 |
| Macedonia | 1.3% | 0.22 | 0.57 | 0.62 | 0.79 |
| Iraq | 1.7% | -0.75 | -0.21 | 0.29 | -0.05 |
| Montenegro | 2.0% | 0.34 | 0.91 | 1.02 |  |
| Moldova | 3.6% | 0.12 | 0.24 | 0.26 | 0.54 |
| Panama | 4.7% |  |  |  | 0.11 |
| Kyrgyzstan | 5.1% | -0.56 | 0.09 | 0.52 |  |
| Algeria | 5.3% | -0.30 | 0.17 | 0.46 | 0.02 |
| Suriname | 5.4% | -0.30 | -0.29 | -0.19 | 0.08 |
| Vietnam | 5.5% | -0.94 | -0.59 | -0.10 | 0.29 |
| Belize | 10.4% | -0.88 | -0.27 | 0.26 | 0.64 |
| Tunisia | 13.6% | -0.12 | 0.51 | 0.79 | 0.24 |
| Guyana | 16.7% | -0.38 | -0.25 | -0.05 |  |
| Kosovo | 29.1% | 0.02 | 0.03 | 0.03 |  |
| Togo | 29.7% | -1.28 | -0.95 | -0.33 | -0.46 |
| Sierra Leone | 36.3% | -1.56 | -0.87 | 0.10 | -0.60 |
| Ghana | 44.2% | -1.08 | -0.90 | -0.42 | -0.06 |
| Sudan | 50.5% | -1.36 | -1.33 | -0.84 |  |
| Central African Republic | 51.6% | -1.42 | -1.03 | -0.30 | -0.62 |
| Nepal | 57.1% | -1.47 | -1.30 | -0.64 |  |
| Nigeria | 59.0% | -1.31 | -1.03 | -0.38 | -0.16 |
| Swaziland | 61.6% | -1.03 | -0.03 | 0.79 | 0.03 |
| Lao | 67.4% | -1.74 | -1.30 | -0.43 | 0.27 |
| Zimbabwe | 67.8% | -1.25 | -0.72 | -0.03 | -0.30 |
| Congo DRC | 77.7% | -1.54 | -1.02 | -0.21 | -0.45 |
| Chad | 80.1% | -1.53 | -1.36 | -0.66 | -0.83 |
| Guinea Bissau | 80.8% | -1.25 | -0.89 | -0.29 |  |
| Malawi | 95.0% | -1.63 | -0.83 | 0.10 |  |
| Total | 34.7% | -1.01 | -0.58 | -0.01 | -0.03 |
