# Supplementary figures and images for: Associations between birth registration and early child growth and development: evidence from 31 low- and middle-income countries
Source: BMC Public Health. 2018 May 30;18:673. doi: 10.1186/s12889-018-5598-z (PMC5977554; doi:10.1186/s12889-018-5598-z)

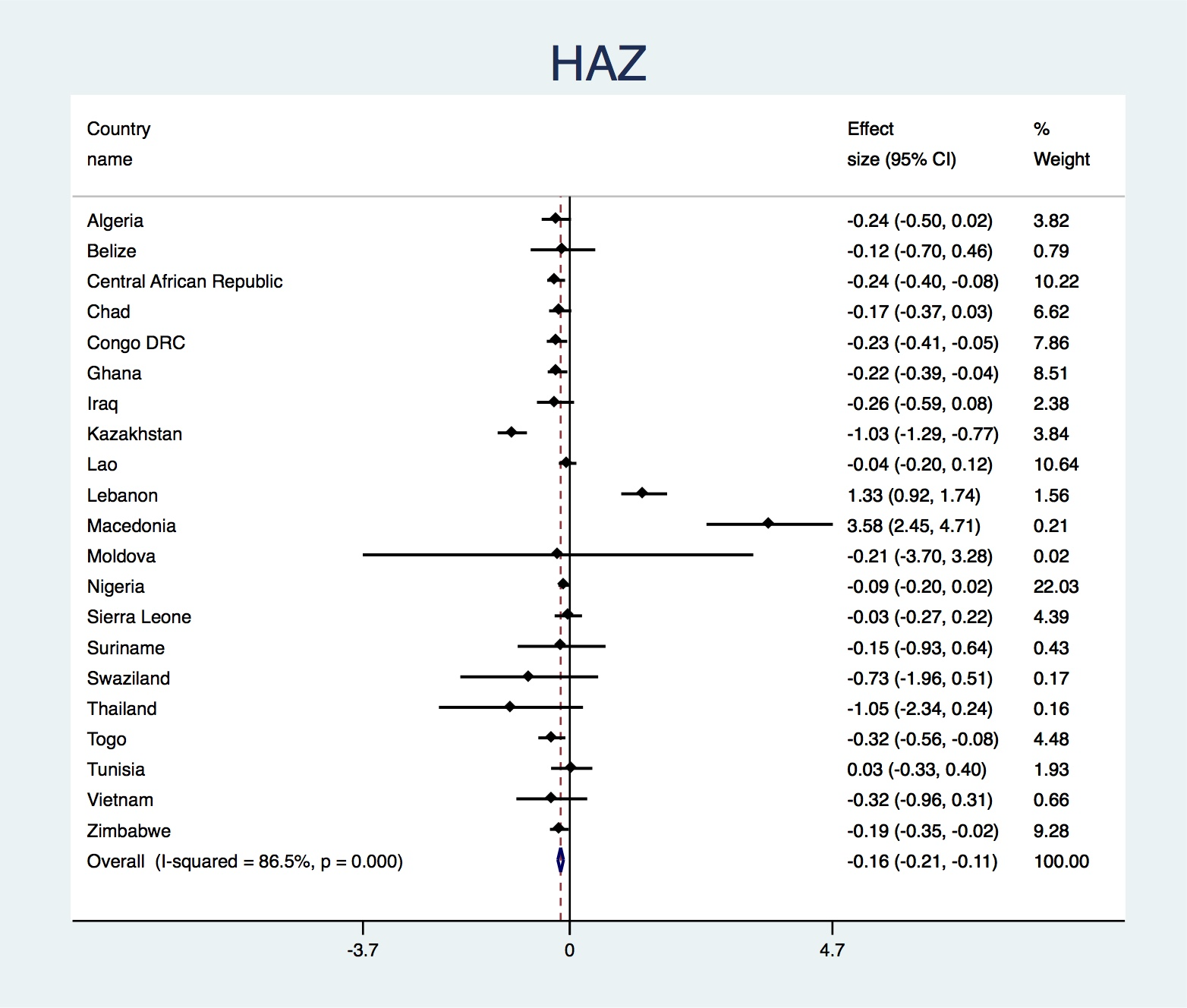

Supplement: Supplementary file 2 — Figure of pooled and country-specific associations between no birth certificate and HAZ among children aged 36–59 months based on meta-regression model. (DOCX 718 kb) [file 12889_2018_5598_MOESM2_ESM.docx]

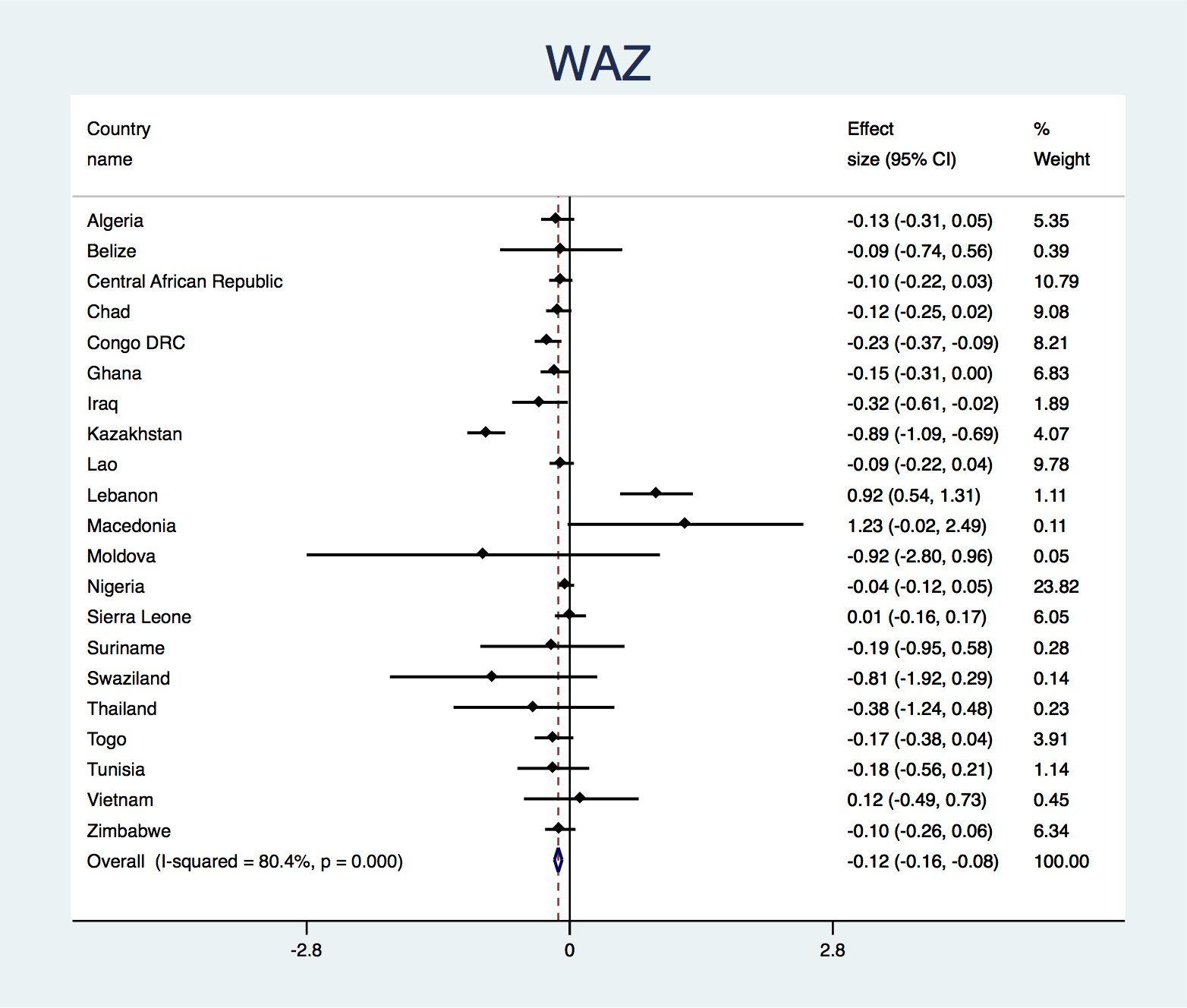

Supplement: Supplementary file 3 — Figure of pooled and country-specific associations between no birth certificate and WAZ among children aged 36–59 months based on meta-regression model. (DOCX 732 kb) [file 12889_2018_5598_MOESM3_ESM.docx]

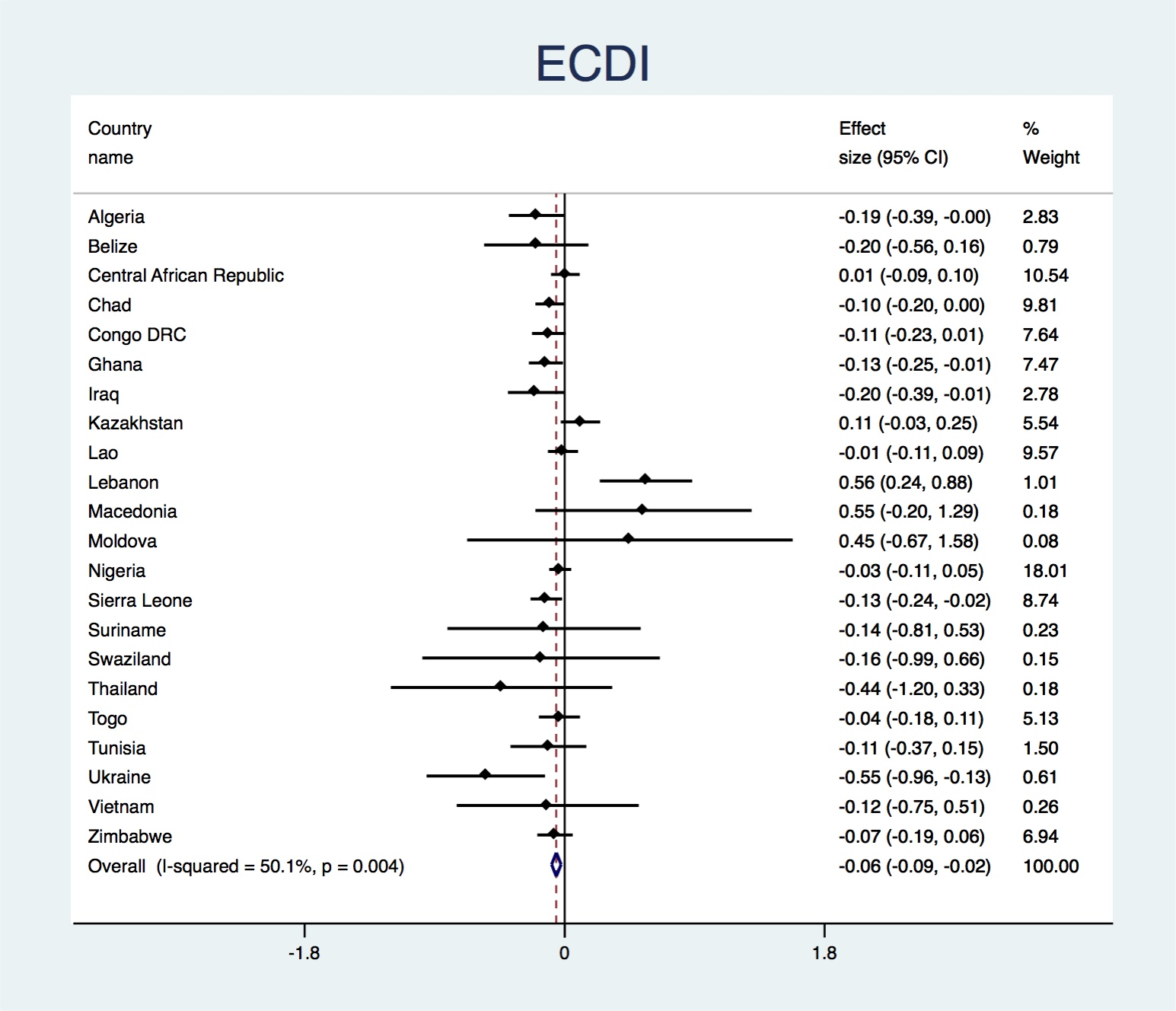

Supplement: Supplementary file 4 — Figure of pooled and country-specific associations between no birth certificate and ECDI z-score among children aged 36–59 months based on meta-regression model. (DOCX 749 kb) [file 12889_2018_5598_MOESM4_ESM.docx]

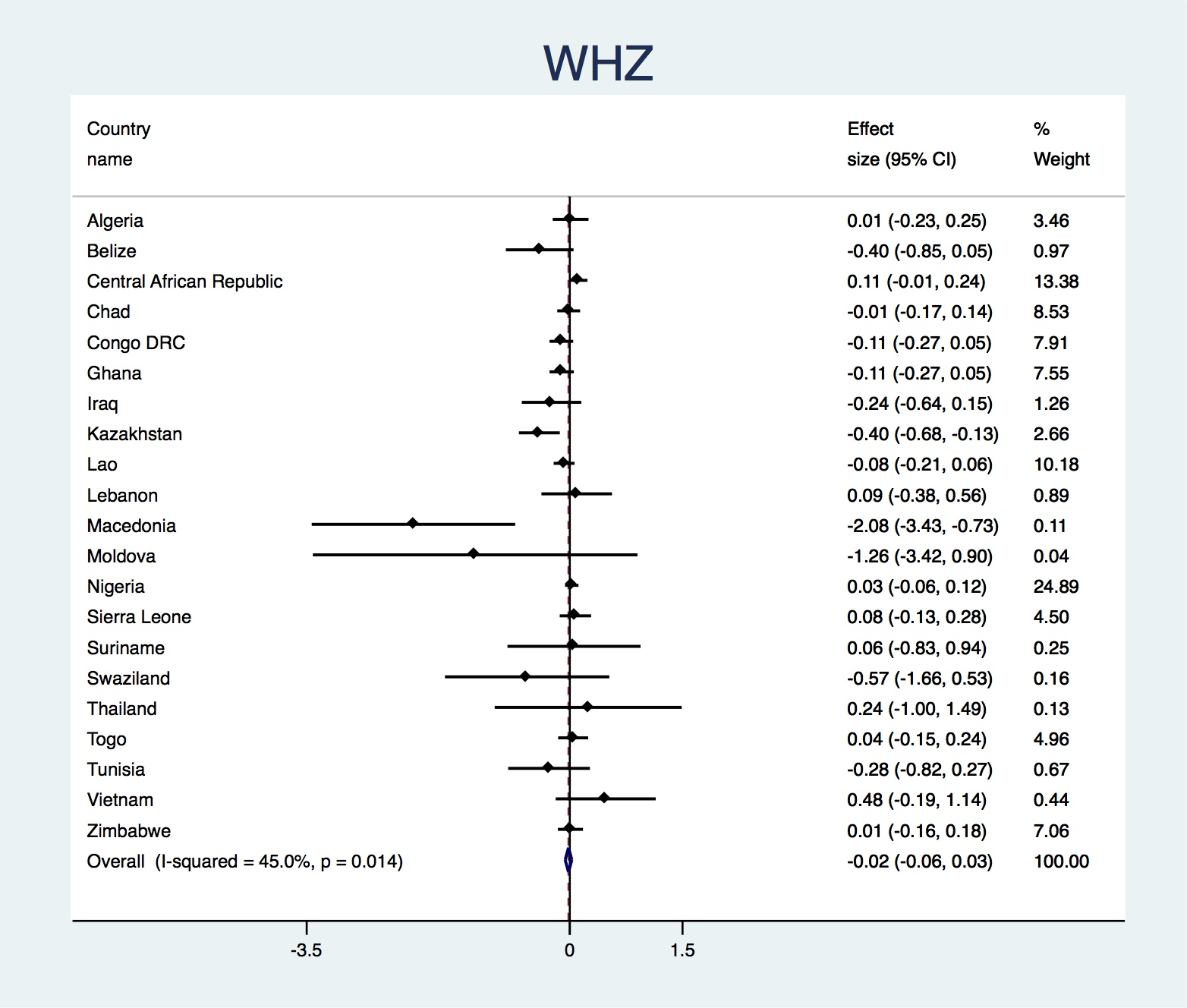

Supplement: Supplementary file 5 — Figure of pooled and country-specific associations between no birth certificate and WHZ among children aged 36–59 months based on meta-regression model. (DOCX 715 kb) [file 12889_2018_5598_MOESM5_ESM.docx]
