# Supplementary material for: Associations between birth registration and early child growth and development: evidence from 31 low- and middle-income countries
Source: BMC Public Health. 2018 May 30;18:673. doi: 10.1186/s12889-018-5598-z (PMC5977554; doi:10.1186/s12889-018-5598-z)
Supplement: Supplementary file 6 — Sensitivity analysis of the associations between no birth certificate and HAZ and WAZ for children aged 0–35 months; and stratified by child age groups (12 month age groups). (DOCX 79 kb) [file 12889_2018_5598_MOESM6_ESM.docx]

| **S2 Table. Sensitivity analysis of the associations between no birth certificate and HAZ and WAZ for children aged 0-35 months; and stratified by child age groups (12 month age groups)** | | | | | | | | | |
| --- | --- | --- | --- | --- | --- | --- | --- | --- | --- |
|  | HAZ | | | | WAZ | | | | |
|  | 0-35 mo (*n* = 100,496) | By age groups | | | | 0-35 mo (*n* = 102,126) | By age groups | | |
|  |  | 0-11 mo (*n* = 31,997) | 12-23 mo (*n* = 34,494) | 24-35 mo (*n* = 34,005) | |  | 0-11 mo (*n* = 32,718) | 12-23 mo (*n* = 34,973) | 24-35 mo (*n* = 34,435) |
| β of no birth certificate | -0.08*** | -0.04 | -0.07* | -0.15*** | | -0.04** | -0.03 | -0.04 | -0.11*** |
| 95% CI | (-0.11, -0.05) | (-0.10, 0.01) | (-0.13, -0.02) | (-0.20, -0.09) | | (-0.07, -0.01) | (-0.08, 0.02) | (-0.08, 0.01) | (-0.15, -0.07) |
| HAZ: height-for-age z-scores; mo: months; WAZ: weight-for-age z-scores.  Notes: The table presents unweighted standardized mean differences in child growth outcomes for children aged 0-35 months in 31 countries who did not have a birth certificate. Models adjusted for child age, sex, maternal age, maternal education, paternal education, household wealth quintiles, urban/rural residency, and vaccines. Model additionally controls for local area characteristics and country fixed effects (Model 4). All standard errors were clustered at the PSU level. | | | | | | | | | |
